# Evaluation of World Population-Weighted Effective Dose due to Cosmic Ray Exposure

**DOI:** 10.1038/srep33932

**Published:** 2016-09-21

**Authors:** Tatsuhiko Sato

**Affiliations:** 1 Japan Atomic Energy Agency, Nuclear Science and Engineering Center, Research Group for Radiation Transport Analysis, Tokai, Ibaraki, 319-1195, Japan

## Abstract

After the release of the Report of the United Nations Scientific Committee of the Effects of Atomic Radiation in 2000 (UNSCEAR2000), it became commonly accepted that the world population-weighted effective dose due to cosmic-ray exposure is 0.38 mSv, with a range from 0.3 to 2 mSv. However, these values were derived from approximate projections of altitude and geographic dependences of the cosmic-ray dose rates as well as the world population. This study hence re-evaluated the population-weighted annual effective doses and their probability densities for the entire world as well as for 230 individual nations, using a sophisticated cosmic-ray flux calculation model in tandem with detailed grid population and elevation databases. The resulting world population-weighted annual effective dose was determined to be 0.32 mSv, which is smaller than the UNSCEAR’s evaluation by 16%, with a range from 0.23 to 0.70 mSv covering 99% of the world population. These values were noted to vary with the solar modulation condition within a range of approximately 15%. All assessed population-weighted annual effective doses as well as their statistical information for each nation are provided in the supplementary files annexed to this report. These data improve our understanding of cosmic-ray radiation exposures to populations globally.

Cosmic rays are one of the major sources of natural radiation exposure to humans. In the 2000 Report of the United Nations Scientific Committee of the Effects of Atomic Radiation (UNSCEAR2000)^1^, the annual effective dose due to cosmic ray exposure averaged over the world’s population was evaluated to be 0.38 mSv, excluding contributions from cosmogenic radionuclides, with range from 0.3 to 2 mSv. This conclusion was maintained in the most recent UNSCEAR report addressing cosmic-ray exposures (UNSCEAR2008)^2^. However, the evaluated doses were determined from approximate projections of altitude and geographic dependences of the cosmic-ray dose rates as well as the world population. For example, population-averaged effective doses for the directly ionizing and neutron components were simply determined from corresponding data at sea level by applying altitude-weighting factors of 1.25 and 2.5, respectively. Note that the term “directly ionizing component” used in UNSCEAR2000 includes contributions from photon exposure; as such, the same terminology is utilized in this study for consistency.

A more detailed investigation of population doses due to cosmic ray exposure was performed by Chen *et al*.^3^. They estimated annual effective doses for more than 1,500 communities across Canada, using the PHITS-based Analytical Radiation Model in the Atmosphere (PARMA) version 2.0^4^,^5^, and concluded that the population-weighted Canadian average dose was 0.31 mSv. PARMA facilitates instantaneous estimation of terrestrial cosmic ray fluxes at any time and location within the atmosphere by supplying atmospheric depth, *d*, in g/cm^2^; vertical cut-off rigidity, *r*_c_, in GV; and solar modulation index, *W*. In addition, it can consider the variation of albedo neutron fluxes due to the difference of water densities in the ground. Thus, it is an ideal tool for analyzing cosmic ray dose rates at ground level for various locations.

In this study, the population-weighted annual effective doses due to cosmic ray exposure for 230 national and sub-national administrative units (simply referred to as “nations” hereafter) were evaluated using a recent version of PARMA^6^. Within the evaluation, the population of each nation as a function of latitude and longitude was obtained from the Gridded Population of the World version 3 (GPW3)^7^, whereas the mean elevations within each grid of GPW3 were calculated from the global digital elevation model GTOPO30^8^. The world population-weighted annual effective dose as well as its probability density were also derived from the analysis. The results of the subject evaluation together with the discussion of their potential ranges and uncertainties due to variations in solar modulation, ground conditions, and building shielding effects are presented below.

## Calculation Procedures

GPW3 provides the population counts of 230 nations by 2.5 arc-minute grid cells for the years 1990, 1995, and 2000; the total populations for each nation were correspondingly adjusted to United Nations (UN) population estimates. The database of the population counts for the year 2000 was selected in this study. Note that the 1990 database was also examined for this assessment to analyze the influence of temporal variations of population counts. Consequently, it was observed that the calculated results are essentially unaffected by the year selected. The mean elevations of each grid were calculated by GTOPO30, which is a global digital elevation model with a horizontal grid spacing of 30 arc-seconds. The calculated elevations were subsequently converted to atmospheric depth, *d*, using US Standard Atmosphere 1976. The vertical cut-off rigidity, *r*_c_, of each grid was taken from a worldwide cut-off rigidity map segmented by a 1-degree grid developed using MAGNETOCOSMICS^9^.

**Figure 1:**
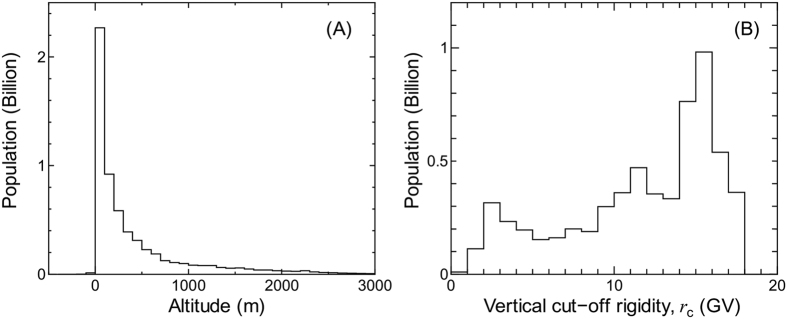
World population distributions as a function of (**A**) altitude or (**B**) vertical cut-off rigidity. These data are obtained from GPW3 coupled with GTOPO30, or the worldwide cut-off rigidity map, respectively.

Figure 1 shows the world population distributions as a function of altitude or vertical cut-off rigidity obtained from GPW3, coupled with GTOPO30 or the worldwide cut-off rigidity map, respectively. It is seen in Panel (A) that more than half of the world population lives on land with altitudes below 200 m. In contrast, as for *r*_c_ dependence, the world’s population is rather widely distributed, particularly at lower and higher *r*_c_ regions. The peaks observed around *r*_c_ = 2 and 15 GV are attributable to the large populations in Europe and North America, and South and Southeast Asia, respectively.

By supplying the evaluated atmospheric depth and vertical cut-off rigidity, the cosmic ray fluxes of neutrons, protons, helium ions, positive and negative muons, electrons, positrons, and photons at ground level of each GPW3 grid were calculated using PARMA3.0. Note that the model can also calculate the cosmic ray fluxes of ions with a charge up to 28, but their contributions are negligible at ground level and were thus disregarded in this study. PARMA3.0 comprises numerous analytical functions with parameters whose numerical values were fitted to reproduce the results of extensive air shower simulations performed by the Particle and Heavy Ion Transport code System (PHITS)^10^. The accuracy of the model was well verified by various experimental data such as particle fluxes, radiation doses, and count rates of ground level neutron monitors^6^. For example, PARMA3.0 can reproduce measured cosmic-ray dose rates at 24 locations over wide ranges of altitudes and vertical cut-off rigidities mostly within 10%, and the C/E ratio averaged over all locations is 0.977. Thus, we expected that the uncertainty of the calculated world population-weighted effective doses associated with the adoption of PARMA3.0 is less than several percent. PARMA3.0 is available freely, as implemented in an open-access software program EXcel-based Program for Calculating Atmospheric Cosmic ray Spectrum (EXPACS)^11^. It should be mentioned that the PARMA model was recently upgraded to version 4.0 by implementing a function to estimate the angular distributions of terrestrial cosmic-rays^12^, but the omni-directional fluxes calculated by the latest version are nearly identical to the corresponding data obtained from PARMA3.0.

The calculated fluxes were then converted to corresponding effective dose rates using the fluence to effective dose conversion coefficients for the isotropic irradiation geometry specified in International Committee on Radiological Protection (ICRP) Publications 116^13^ and 123^14^. The population-weighted effective dose rates for outdoors were then derived from the aforementioned calculated effective dose rates multiplied by the population within each grid. The absorbed dose rates in air outdoors at ground or sea level were also derived from the calculated cosmic ray fluxes for charged particles using their collision stopping power in dry air. It should be mentioned that the restricted collision stopping power below 10 keV was adopted for converting electron and positron fluxes to corresponding absorbed dose rates to avoid double counting of the contributions of higher energy particles.

In addition to the atmospheric depth and vertical cut-off rigidity, the solar modulation index, *W*, and the water density in the ground, ρ_w_ , must be supplied to PARMA3.0 for calculating cosmic ray fluxes at the ground level. The numerical value of *W* is roughly consistent with the sun spot number, though its actual value is determined from the count rates of several neutron monitors. In this study, an approximated mean value of *W* = 50 after the year 2000 was employed for calculating the mean population-weighted annual effective doses. As for the water density, a typical value for representing ground-level neutron fluxes, i.e. ρ_w_ = 0.20, was employed for the calculation. Furthermore, variations of the population-weighted annual effective doses were assessed by changing *W* = 0 to 150 for representing solar minimum and maximum conditions, respectively, and by changing ρ_w_ = 0 to 1.0 for representing completely dry ground and pure water, respectively.

## Results and Discussion

### Absorbed Dose Rates in Air Outdoors

**Figure 2:**
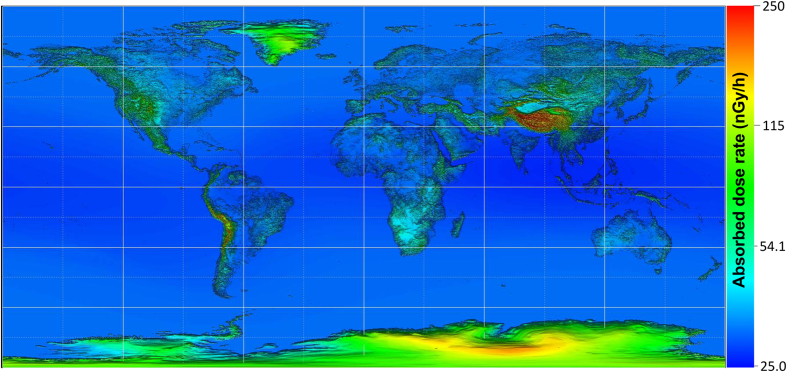
Calculated absorbed dose rates owing to cosmic-ray exposure in outdoor air at ground or sea level for 2.5 arc-minute grid cells. The colors are assigned in a logarithmic scale of the dose rates. This figure was generated by AVS/Express Developer Edition Version 8.2 (http://www.avs.com/solutions/express/).

Figure 2 shows the map of calculated absorbed dose rates in air outdoors due to cosmic ray exposure at ground or sea level for 2.5 arc-minute grid cells. The *W* index was set to 50 in this calculation. It can be seen that the absorbed dose rates increase at higher latitude and altitude regions. The mean absorbed dose rate for the entire world including sea areas is 33.7 nGy/h, while that averaged over land areas is 42.8 nGy/h. The highest dose rate is 502 nGy/h observed around Mt. Everest (elevation = 7,987 m; *r*_c_ = 14.5 GV), while the lowest dose rate is around 26.8 nGy/h observed around Car Nicobar Island in the Indian Ocean (elevation = 0 m; *r*_c_ = 17.7 GV). Note that the elevation of the highest dose point is lower than that at the summit of Everest, 8,848 m, owing to the employment of the mean elevation of 2.5 arc-minute grid cells in the calculation.

### Population-Weighted Annual Effective Dose

Table 1 summarizes the population-weighted annual effective doses for the entire world and the nations with populations over 100 million, classified according to contributions from particles incident upon the human body. Note that the proton and electron data include contributions from helium ions and positrons, respectively. The standard deviation as well as the minimum and maximum of the total annual effective doses are also tabulated. The complete data set for all nations is presented in Supplementary File S1.

It is found from the table that the world population-weighted annual effective dose obtained from this study, 0.340 mSv, is smaller than the corresponding data obtained from UNSCEAR2000, 0.46 mSv, by approximately 26%. Note that the “well-known” UNSCEAR assessed value of 0.38 mSv was determined by considering a building shielding factor of 0.8 and an indoor occupancy factor of 0.8. The three main differences between this study’s and UNSCEAR’s evaluations are the following:

1. The altitude-weighted factors employed in UNSCEAR2000 were probably overestimated because they were determined using an approximate altitude–population distribution. Based on our model, the population-weighted annual effective doses calculated employing an “ideally flat” Earth, i.e. assuming the elevation of all land is 0 m, are 0.234 and 0.0390 mSv for the directly ionizing and neutron components, and the corresponding altitude-weighted factors are 1.17 and 1.71, respectively. These values were observed to be smaller than the UNSCEAR2000 evaluations by approximately 6% and 32%, respectively.
2. The effective doses from directly ionizing component at sea level employed in UNSCEAR2000, which were 30 and 32 nSv/h for latitudes below and above 30°, respectively, maintain large degrees of uncertainty. According to UNSCEAR1988^15^, these values were simply taken from experimental absorbed dose rates in outdoor air measured at latitudes of approximately 20°N and 40°N in the 1970’s^16^; thus, they are expected to be different from the effective doses from the directly ionizing component. The absorbed dose rates in outdoor air at sea level calculated by our model gradually increase from 26.8 to 31.8 nSv/h with decrease in *r*_c_. These values were observed to be higher than the corresponding effective dose rates by approximately 9%.
3. The definitions of the effective dose employed in UNSCEAR2000 and this study are different, which are based on ICRP Publications 60^17^ and 103^18^, respectively. An important change introduced in ICRP 103 is that the radiation weighting factors for neutrons and protons are reduced from the corresponding values defined in ICRP 60, and consequently, the fluence to effective dose conversion coefficients likewise decreased for those particles. Our calculation suggests that the use of ICRP 60 instead of ICRP 103 results in an increase in population-weighted annual effective dose from neutrons and protons by factors of 1.14 and 2.5, respectively, resulting in an increase in the total aggregate dose by approximately 4%.

In general, the population-weighted annual effective dose decreases for nations located near the equator with a lower mean elevation, such as Bangladesh as listed in Table 1. The nation having the highest population-weighted annual effective dose (0.921 mSv) is Bolivia, while those having the lowest value (0.245 mSv) are Maldives and Singapore. Note that the majority of Bolivians reside in cities located at high altitude such as La Paz. For nations having a lower population-weighted annual effective dose, the muon contribution reaches up to 70% of the total, while it decreases with an increase in overall annual effective dose because the doses from other particles are more sensitive to global conditions. For example, neutrons and muons have nearly equal contributions to the total for the Bolivian case. The maximum dose in a nation is generally observed at the highest elevation point in which people reside because *r*_c_ values usually do not vary much within a nation.

It is practically impossible at the present juncture to directly verify the accuracy of calculated population-weighted effective doses by measurement. One tangible source that may be utilized in a verification-type process, however, are the results obtained from the nationwide measurements of cosmic ray dose rates throughout Japan^19^. The measured neutron effective dose rate averaged over 240 points in Japan during 2002 to 2005 was 4.8 nSv/h, while the calculated population-weighted neutron effective dose rate in Japan was 4.96 nSv/h. Considering the difference between the distributions of measured points and the Japanese population, this numerical agreement is viewed as quite satisfactory.

### Population Variance of the Annual Effective Dose

**Figure 3:**
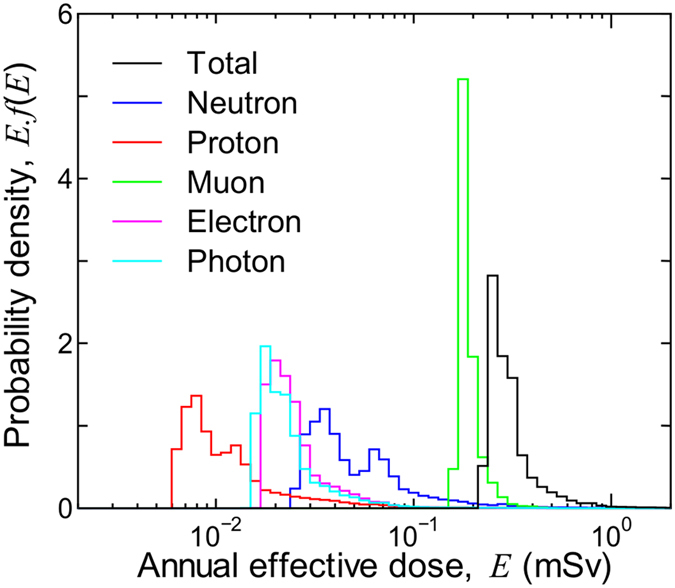
Probability densities of annual effective doses of the world population, *E*.*f*(*E*), classified according to contributions from particles incident upon the human body. Building shielding effects are not considered in the data.

Figure 3 shows the probability densities of the annual effective doses of the world population, *E*.*f*(*E*), classified according to contributions from particles incident upon the human body. The integral of *f*(*E*) with respect to *E* is normalized to 1.0. The standard deviation of the total, neutron, proton, muon, electron, and photon doses are 46.1%, 105%, 132%, 14.5%, 63.3%, 73.2% of their population-weighted values, respectively. As expected from the discussion above, the variance of the muon doses is rather small in comparison with those for other particles because muon dose is less sensitive to global conditions. The probability densities of the neutron and proton doses have two peaks, which are primarily attributed to the large populations in high and low *r*_c_ regions, as shown in Fig. 1(B). The complete data set of the probability densities of the annual effective doses for all nations is presented in Supplementary File S2.

### Variation with Solar Modulation and Ground Condition

The world population-weighted annual effective doses calculated for the solar minimum and maximum conditions, i.e. *W* = 0 and 150, are 0.348 and 0.302 mSv, respectively. The daily *W* value occasionally drops below 0 or becomes greater than 150, but the minimum and maximum values of its annual mean values are 5.1 and 129.8 over the past 65 years, which were observed in 2009 and 1990, respectively. Thus, it was ultimately determined that world population-weighted annual effective doses generally vary with solar modulation within a margin of approximately 15%.

In PARMA3.0, neutron fluxes are influenced by ground conditions because more neutrons are reflected by dry ground than wet ground as neutrons are absorbed by hydrogen atoms. The world population-weighted annual effective doses due to neutron exposure calculated by setting ρ_w_ = 0 and 1.0 were 0.0817 and 0.0590 mSv, respectively. This variation can be regarded as reasonable considering the fact that neutron doses seasonally changed by approximately 14% due to snow covering ground^20^. As observed in Table 1, the neutron contribution is approximately 20% of the total dose, and consequently, the ground condition can change the total dose by up to 6%. However, the assumption of ρ_w_ = 0 or 1.0 for all populations is obviously unrealistic; hence, the uncertainty of world population-weighted effective doses is expected to be less than a few percent owing to the ambiguity of ground conditions.

### Building Shielding and Indoor Occupancy Factors

In general, cosmic ray dose rates inside a building are smaller than those outdoors. Thus, building shielding and indoor occupancy factors should be considered for estimating the most likely population-weighted effective dose. UNSCEAR2000 summarized that observed building shielding factors ranged from close to 1 for a small wooden house to 0.4 for the lower levels of a substantial concrete building. As such, a universally representative value of 0.8 for the shielding factor was thus asserted in the evaluation for the most likely population-weighted effective dose. However, this value may be considered too low, as the majority of cosmic ray doses comprise muon contributions that are hardly shielded by conventional buildings. In addition, only a small portion of people live in substantial concrete buildings, particularly in developing countries.

The shielding factor was therefore re-evaluated in this present study by introducing the assumption that the building wall can be represented by the same mass thickness of dry air in terms of the radiation shielding effect. With the exception of neutron doses, this assumption is basically sound because the building materials are generally composed of lighter atoms similar to dry air. For neutron doses this assumption may underestimate the shielding effect because hydrogen atoms in building materials, which are not present in dry air, are very important for neutron shielding. Under this assumption, the effective dose rates inside a building with wall mass thickness *t* can be calculated by PARMA3.0, supplying “*d* + *t*” instead of “*d*” as the atmospheric depth.

**Figure 4:**
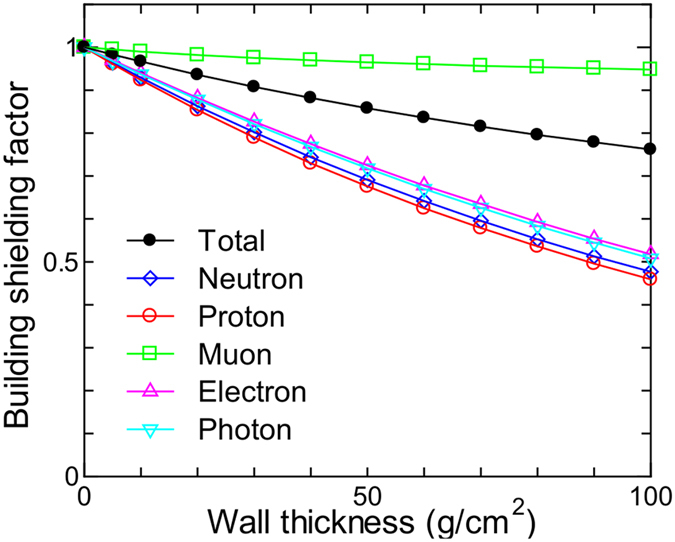
Calculated building shielding factors to be multiplied with world population-weighted effective doses. Lines are just for eye guide.

Figure 4 shows the calculated building shielding factors to be multiplied with the world population-weighted effective doses for each particle contribution as a function of the wall mass thickness. It is seen that the building shielding factors for muon doses are very close to 1.0 even for thicker walls, while those for other particles rapidly decrease with increasing wall thickness. In order to achieve a building shielding factor of 0.8 for the total dose, the wall thickness should be approximately 80 g/cm^2^. Considering the fact that the sum of the roof and ceiling thicknesses of a typical Japanese concrete dwelling is approximately 30 g/cm^2^, this value is too thick as a representative value for conventional homes across the world. We therefore propose 30 g/cm^2^ as the representative wall thickness for estimating the most likely value of the world population-weighted effective dose, and 5 and 80 g/cm^2^ for estimating its possible maximum and minimum values, respectively. These values correspond to shielding factors of 0.91, 0.98 and 0.80, respectively.

Based on this proposal, and assuming an indoor occupancy factor of 0.8 as per UNSCEAR2000, the world population-weighted annual effective dose after considering shielding by buildings is 0.32 mSv, with uncertainties of approximately +6% and −9% due to the choice for the shielding factor. The shielding factor will be lower for nations with higher population-weighted doses, e.g. 0.85 for the Bolivian case, because the contribution of the muon dose to the total becomes smaller with an increase of altitude owing to more dramatic increase of the dose from other particles such as neutrons at higher altitudes. Table 2 summarizes the population-weighted annual effective doses after considering building shielding effects for the entire world and the nations with populations over 100 million. The world minimum and maximum values are 0.23 and 6.1 mSv, which are observed in India and Nepal, respectively, but 99% of world population are expected to have the annual effective dose within the range between 0.23 and 0.70 mSv. The complete data sets after considering building shielding effects for all nations are also presented in Supplementary Files S1 and S2.

## Conclusions

The population-weighted annual effective doses and their probability densities for the entire world as well as each nation were evaluated using PARMA3.0 coupled with GPW3 and GTOPO30 databases. The evaluated world population-weighted annual effective dose before and after considering building shielding effects are 0.340 and 0.32 mSv, which are smaller than the corresponding data evaluated in UNSCEAR2000 by approximately 26% and 16%, respectively. These values generally vary with solar conditions within approximately 15% and maintain an uncertainty of a few percent owing to ground condition ambiguities. Additional +6% and −9% uncertainties are inherent within the data after considering building shielding effects owing to crude estimates of representative wall thicknesses of conventional dwellings. More detailed analyses for estimating building shielding factors such as cosmic ray transport simulations inside various types of housing are necessary to reduce such uncertainties. Nonetheless, the conclusion about UNSCEAR’s result, 0.38 mSv, being too high will not be affected by such analyses, because this value is greater than our evaluated value before considering building shielding effects. A similar calculation based on other models and tools is also desirable to confirm the accuracy of the presented results.

## Additional Information

**How to cite this article**: Sato, T. Evaluation of World Population-Weighted Effective Dose due to Cosmic Ray Exposure. *Sci. Rep.* **6**, 33932; doi: 10.1038/srep33932 (2016).

## Supplementary Material

Supplementary File S1

Supplementary File S2

## Figures and Tables

**Table 1:** Population-weighted annual effective doses before considering building shielding effects for the entire world and nations with populations over 100 million, classified according to contributions from particles incident upon the human body.

ISO3166 alpha-3 code	Population at year 2000	Annual effective dose before considering building shielding effects (mSv)								
		Neutron	Proton	Muon	Electron	Photon	Total	σ	Minimum	Maximum
World	6033968093	6.66E-02	1.57E-02	2.02E-01	2.88E-02	2.69E-02	3.40E-01	1.57E-01	2.42E-01	6.96E+00
CHN	1253492596	6.31E-02	1.53E-02	2.03E-01	2.89E-02	2.69E-02	3.37E-01	1.67E-01	2.47E-01	5.81E+00
IND	1008934399	4.31E-02	1.06E-02	1.88E-01	2.42E-02	2.24E-02	2.88E-01	1.33E-01	2.42E-01	6.38E+00
USA	283200526	8.49E-02	1.69E-02	2.06E-01	2.82E-02	2.65E-02	3.62E-01	9.65E-02	2.67E-01	5.84E+00
IDN	212056274	3.76E-02	9.04E-03	1.85E-01	2.25E-02	2.06E-02	2.74E-01	4.30E-02	2.42E-01	1.69E+00
BRA	170401328	5.78E-02	1.32E-02	2.05E-01	2.80E-02	2.55E-02	3.30E-01	4.90E-02	2.67E-01	8.05E-01
RUS	145489933	7.95E-02	1.55E-02	2.04E-01	2.73E-02	2.57E-02	3.52E-01	6.53E-02	2.89E-01	2.77E+00
PAK	141256130	5.99E-02	1.49E-02	2.01E-01	2.80E-02	2.63E-02	3.30E-01	1.95E-01	2.55E-01	5.17E+00
BGD	137439020	3.03E-02	7.11E-03	1.80E-01	1.89E-02	1.72E-02	2.54E-01	2.25E-03	2.49E-01	3.16E-01
JPN	127094637	4.35E-02	9.68E-03	1.94E-01	2.29E-02	2.07E-02	2.91E-01	3.54E-02	2.55E-01	9.74E-01
NGA	113861481	4.17E-02	9.83E-03	1.90E-01	2.43E-02	2.22E-02	2.88E-01	2.43E-02	2.59E-01	5.30E-01

The standard deviation, σ, as well as the minimum and maximum of the total annual effective doses are also given.

**Table 2:** Population-weighted annual effective doses after considering building shielding effects for the entire world and nations with populations over 100 million, classified according to contributions from particles incident upon the human body.

ISO3166 alpha-3 code	Population at year 2000	Annual effective dose after considering building shielding effects (mSv)								
		Neutron	Proton	Muon	Electron	Photon	Total	σ	Minimum	Maximum
World	6033968093	5.6E-02	1.3E-02	2.0E-01	2.5E-02	2.3E-02	3.2E-01	1.3E-01	2.3E-01	6.1E+00
CHN	1253492596	5.3E-02	1.3E-02	2.0E-01	2.5E-02	2.3E-02	3.1E-01	1.4E-01	2.4E-01	5.0E+00
IND	1008934399	3.6E-02	8.9E-03	1.9E-01	2.1E-02	1.9E-02	2.7E-01	1.1E-01	2.3E-01	5.5E+00
USA	283200526	7.1E-02	1.4E-02	2.0E-01	2.4E-02	2.2E-02	3.3E-01	8.2E-02	2.5E-01	4.9E+00
IDN	212056274	3.2E-02	7.6E-03	1.8E-01	1.9E-02	1.8E-02	2.6E-01	3.7E-02	2.3E-01	1.5E+00
BRA	170401328	4.9E-02	1.1E-02	2.0E-01	2.4E-02	2.2E-02	3.1E-01	4.2E-02	2.5E-01	7.2E-01
RUS	145489933	6.6E-02	1.3E-02	2.0E-01	2.3E-02	2.2E-02	3.2E-01	5.5E-02	2.7E-01	2.4E+00
PAK	141256130	5.1E-02	1.2E-02	2.0E-01	2.4E-02	2.2E-02	3.1E-01	1.7E-01	2.4E-01	4.5E+00
BGD	137439020	2.6E-02	6.0E-03	1.8E-01	1.6E-02	1.4E-02	2.4E-01	1.9E-03	2.4E-01	2.9E-01
JPN	127094637	3.7E-02	8.1E-03	1.9E-01	1.9E-02	1.7E-02	2.7E-01	3.0E-02	2.4E-01	8.6E-01
NGA	113861481	3.5E-02	8.2E-03	1.9E-01	2.1E-02	1.9E-02	2.7E-01	2.1E-02	2.5E-01	4.8E-01

The standard deviation, σ, as well as the minimum and maximum of the total annual effective doses are also given.
